# Robotic adrenalectomy: comparison of DaVinci, HUGO™-RAS and Versius^®^ platforms—a preliminary retrospective analysis

**DOI:** 10.1007/s13304-025-02414-8

**Published:** 2025-09-18

**Authors:** Priscilla Francesca Procopio, Francesco Pennestrì, Pierpaolo Gallucci, Antonio Laurino, Annamaria Martullo, Carmela De Crea, Marco Raffaelli

**Affiliations:** 1 U.O.C. Chirurgia Endocrina e Metabolica, Fondazione Policlinico Universitario Agostino Gemelli IRCCS, L.Go A. Gemelli 8, 00168 Rome, Italy; 2 Centro di Ricerca in Chirurgia delle Ghiandole Endocrine e dell‘Obesità, Università Cattolica del Sacro Cuore, Rome, Italy

**Keywords:** Robotic adrenalectomy, DaVinci, HUGO™-RAS, Versius^®^, Postoperative complications, Operative time

## Abstract

Robot-assisted adrenalectomy (RAA) has emerged as an advantageous approach in challenging cases and suspicious lesions, although high costs represent the main drawback to its broader application. Besides Da-Vinci, new platforms have been recently launched on the market and need to be validated in clinical practice. DaVinci, HUGO™-RAS and Versius^®^ platforms were introduced in our center in 2012, 2022, and 2024, respectively. We aimed to compare the perioperative outcomes of these robotic platforms. Among 730 adrenalectomies (2012–2024), 149 (20.4%) RAAs were performed. All procedures performed by means of HUGO™-RAS and Versius^®^ platforms were compared with similar procedures (in terms of patient’s and lesion’s features) performed with the DaVinci technology in the same period (2022–2024). Ten patients were included in each group. Patients’ and lesions’ features were similar. Median BMI and lesions' size were 27.9, 24.9, and 26.1 kg/m^2^ and 42.5, 42.5, and 32.5 mm in DaVinci, HUGO™-RAS, and Versius^®^ groups, respectively (*p* = 0.360, *p* = 0.236). The groups were comparable for docking time, console time, and operative time (5 vs. 5 vs. 7 min, *p* = 0.059, 58.5 vs. 58 vs. 39 min, *p* = 0.393, 109.5 vs. 110.5 vs. 104.1 min, *p* = 0.668, respectively). No conversion or perioperative complications were registered. Postoperative hospital stays were similar (2 days in all groups, *p* = 0.629). RAA, whatever platform is used, confirmed to be a safe and effective approach, potentially expanding indications for minimally invasive adrenalectomy. Even though new platforms have been applied in less demanding cases in our learning curve phase, the introduction of new different platforms may lead to a costs reduction, thus to a broader diffusion of RAA.

## Introduction

Minimally invasive adrenalectomy has become the gold standard for the treatment of most adrenal lesions, with the well-known advantages in terms of shorter postoperative recovery and hospital stay and lower complications rates [1–3].

The introduction of robotic platforms in clinical practice aimed to further improve precision and ergonomics of minimally invasive surgery, thus overcoming recognized restrictions, such as unstable camera holding and vision and limited movement dexterity [4–7]. In this context, robot-assisted adrenalectomy (RAA) proved to be a safe and feasible option for the treatment of adrenal lesions in several centers [1–3, 8]. Indeed, the application of robotic technology has been reported to be potentially convenient in challenging cases, such as obese patients, hyperfunctioning and > 6 cm lesions and previous abdominal surgery [9–11]. However, randomized study investigating the unequivocal advantages of robotic approach to adrenalectomy are still lacking and to date high costs represent the main drawback to its widespread diffusion in clinical practice [12]. On the other hand, RAA cost-effectiveness and economic sustainability have been described in high-volume centers, especially when robotic technologies have been addressed to the most complex cases [10].

However, it is to highlight that, differently from the established DaVinci system, which is associated with automatic configuration and advanced energy sources, younger platforms still partially lack in such technical advantages. Indeed, both HUGO™-RAS and Versius^®^ technologies are characterized for manual docking, a further trocar is required for the assistant due to the unavailability of specific energy instruments, and indocyanine green has recently been introduced for HUGO™-RAS platform.

Apart from these considerations, the introduction of new technologies, different from the DaVinci systems, is expected to be met with significative and competitive reduction in costs, promoting the availability of robotic platforms worldwide [13, 14] and providing data for further validation of their clinical value.

With the present feasibility study, we aimed to evaluate the safety of RAA performed by means of the more recently introduced robotic platforms, such as HUGO™-RAS and Versius^®^ systems, compared to the consolidated DaVinci Xi platform.

## Materials and methods

RAA was first performed in our center in 2012 by means of DaVinci Si platform (DaVinci—Intuitive Surgical, Sunnyvale, CA, USA) [10]. Afterwards, in 2014, it was displaced by the Xi platform [15]. HUGO™-RAS (HUGO™-RAS—Medtronic, Minneapolis, MN, USA) and Versius^®^ (Versius CMR Surgical, Cambridge, UK) platforms were introduced in 2022 and 2024, respectively.

### Study design

Prospectively collected data from all patients who underwent adrenalectomy in our referral center for endocrine surgery were retrospectively reviewed.

Among all adrenalectomies performed between January 2012 and December 2024, bilateral or subtotal procedures and adrenalectomies performed with laparoscopic, retroperitoneoscopic, or laparotomic approach were excluded from the study.

To achieve a homogeneous population for the study and reduce the potential confounding factors related to the surgical expertise, all procedures performed by means of HUGO™-RAS and Versius^®^ platforms were compared with similar procedures performed with DaVinci technology in the same period (2022–2024).

Perioperative characteristics were evaluated. Patients’ demographic features included BMI, sex as assigned at birth, and age. Other analyzed preoperative variables encompassed lesions’ side and size, hyperfunction, previous abdominal surgery, and ASA score. Intraoperative variables included total operative time, docking time, console time, collisions rates, and intraoperative complications. The considered postoperative features were postoperative stay, postoperative 30 days-complications and readmissions within 30 days, mortality within 30 days, and definitive pathology reports.

Follow-up evaluation was achieved through outpatient consultation or telephonic contact until 31^st^ March 2025.

The study was submitted and approved by the ethical board of our Institution (ID 4853).

The primary outcome of the study was to evaluate the postoperative complications rate of patients who underwent RAAs performed by means of HUGO™-RAS and Versius^®^ systems, comparing them with the same procedures with the DaVinci platform.

The secondary outcomes consisted in the evaluation of the operative time, including total operative time, docking time and console time and intraoperative complications in these patients’ cohort.

### Definitions

Informed consent was administered in all patients.

Preoperative work-up and patients’ selection for surgery were conducted according to international society guidelines and recommendations [16–18].

The operative time is defined as the interval from incision to wound closure (skin to skin). Intraoperative complications were defined as all the events that could potentially cause injury and require unplanned surgical maneuvers. Postoperative complications were defined as any event altering the regular postoperative course and/or delaying discharge, occurring within the 30^th^ postoperative day, with the severity graded according to the Clavien–Dindo classification [19]. Mortality was defined as any intraoperative or postoperative death within 30 days after surgery. Follow-up time is defined as the time interval between the date of the surgical procedure and the date of the last follow-up examination.

### Surgical technique

All robotic procedures were performed via lateral transabdominal approach (LTA). In this series, all procedures were performed by an expert endocrine surgeon (M.R). Details of the surgical procedure have been previously reported [20]. Applications of DaVinci and HUGO™-RAS platforms have been previously described [10, 21]. For what concerns the technical details of Versius^®^ system, this platform consists of four independent arm carts. Each arm is characterized by its own settings and it can be modulated basing on patient’s specific body features. Furthermore, Versius^®^ system can be used by means of a bimodal arm shaping: U-shape may be preferable in case of single quadrant surgery, though its degree range may be limited during ipsilateral working, while Z-shape can serve both in transversal and longitudinal position, thus recommended for multiquadrant approach. In our practice, three robotic arms are used for the surgical procedure, regardless of the lesion site, with the arm for the optic trocar with the Z-shape and the other arms with the U-shape.

Use of a specific robotic technology was chosen basing on the platform availability, surgeon’s preference, and patient’s and lesion’s features. In detail, challenging cases, consisting in a combination of obesity, high lesion size, and/or hyperfunction, and in presence of lesions suspected for adrenocortical carcinoma without preoperative evidence of extra-adrenal extension of the disease, were more frequently addressed to DaVinci system, due to the surgeon’s more consolidated experience with such platform.

### Statistical analysis

Chi-square test was used to compare categorical variables. Continuous variables were expressed as median (interquartile range, IQR). We used ANOVA or Kruskal–Wallis test to compare continuous variables, depending on data distribution of the analyzed population. Statistical analysis was conducted with SPSS 22.0 software for Windows (SPSS Inc, Chicago, III). All analyses were two-tailed, and the threshold for statistical significance was set at *p* < 0.05.

## Results

Among 730 adrenalectomies performed between January 2012 and December 2024, 149 (20.4%) RAAs with LTA approach were performed. These procedures included 129, 10, and 10 adrenalectomies performed by means of DaVinci Xi, HUGO™-RAS, and Versius^®^ platforms, respectively. During the considered time period for the present study (2022–2024), a total of 46 RAAs were performed.

Patients were divided into three groups, according to the specific platform used during surgical procedure.

Out of all the adrenalectomies performed with each platform, ten patients per group were selected due to homogeneous preoperative characteristics (BMI, lesion size, and hyperfunction).

Perioperative variables of the selected population are reported in Table 1. Demographic characteristics were similar among the groups, in terms of sex and age: 5, 4, and 4 male patients vs 5, 6, and 6 female patients and 56 (IQR 52.7–59), 65 (IQR 57.7–74.5), and 61 (IQR 51.7–63.5) years-old patients for DaVinci, HUGO™-RAS, and Versius^®^ platforms (*p* = 0.873, *p* = 0.109), respectively. Median BMI was also comparable among the selected patients, though slightly higher in the DaVinci group: 27.9 (IQR 25-4-30.1) vs 24.9 (IQR 22.6–28.3) and 26.1 (IQR 25.2–19.3) kg/m^2^ for HUGO™-RAS and Versius^®^ patients, respectively (*p* = 0.360). Lesion side was right in 3, 8, and 3 patients and left in 7, 2, and 7 patients for each group, respectively (*p* = 0.035). *Post hoc* analysis confirmed higher rates of right lesions in HUGO™-RAS group: for DaVinci vs HUGO™-RAS *p* = 0.025, for DaVinci vs Versius^®^ *p* = 1, and for HUGO™-RAS vs Versius^®^ *p* = 0.025. For each group, 6, 3, and 4 lesions showed no hyperfunction after preoperative work-up (*p* = 0.711), while in the remaining patients, hyperfunctioning pattern consisted of hypercortisolism (2, 4, and 3 patients, respectively), hyperaldosteronism (1, 0 and 1 patient, respectively), and pheochromocytoma (1, 3, and 2 patients, respectively). Lesions’ size was comparable between the groups, though lower in the Versius^®^ group: 32.5 (IQR 28–42) mm vs 42.5 (IQR 40–56.2) and 42.5 (IQR 37.2–56.2) mm for DaVinci and HUGO™-RAS patients, respectively (*p* = 0.236). No significant differences were registered when previous abdominal surgery was analyzed among the preoperative patients’ variable (*p* = 0.271). ASA score was also comparable among the patients (*p* = 0.535).

**Table 1:** Perioperative variables of patients who underwent robotic adrenalectomy by means of DaVinci, HUGO™-RAS, and Versius^®^ platforms

	DaVinci Xi	HUGO™-RAS	Versius^®^	*p* value
Patients	10	10	10	
Age (years)	56 (52.7–59)	65 (57.7–74.5)	61 (51.7–67.5)	0.109
BMI (kg/m^2^)	27.9 (25.4–30.1)	24.9 (22.6–28.3)	26.1 (25.2–29.3)	0.360
Sex				
Male Female	5 (50%) 5 (50%)	4 (40%) 6 (60%)	4 (40%) 6 (60%)	0.873
Hyperfunction No Cushing Conn Pheo	6 (60%) 2 (20%) 1 (10%) 1 (10%)	3 (30%) 4 (40%) – 3 (30%)	4 (40%) 3 (30%) 1 (10%) 2 (20%)	0.711
Side				
Right Left	3 (30%) 7 (70%)	8 (80%) 2 (20%)	3 (30%) 7 (70%)	0.035 *AvsB = 0.0246 AvsC = 1 BvcC = 0.0246
Size (mm)	42.5 (40–56.2)	42.5 (37.2–56.2)	32.5 (28–42)	0.236
Previous surgery				
No Yes	9 (90%) 1 (10%)	8 (80%) 2 (20%)	6 (60%) 4 (40%)	0.271
ASA score				
2 3	9 (90%) 1 (10%)	7 (70%) 3 (30%)	8 (80%) 2 (20%)	0.535
Docking time (min)	5 (4–6)	5 (5–6.5)	7 (5–8.2)	0.059
Console time (min)	58.5 (38.7–63.5)	58 (33.2–97.5)	39 (33.7–61)	0.393
Operative time (min)	109.5 (78.7–119.5)	110.5 (82–140.2)	104.1 (72.5–143.5)	0.668
Postoperative intensive care				
No Yes		41 (97.6%) 1 (2.4%)	5 (83.3%) 1 (16.7%)	0.101
Collisions	0 (0–0)	0 (0–1)	0 (0–0.2)	0.189
Intraoperative complications				
No Yes	10 (100%) –	10 (100%) –	10 (100%) –	1
Postoperative hospital stay (days)	2 (2–2)	2 (2–3)	2 (2–3)	0.629
Postoperative complications				
No Yes	10 (100%) –	10 (100%) –	10 (100%) –	1
Mortality				
No Yes	10 (100%) –	10 (100%) –	10 (100%) –	1
Final pathology				
Benign Pheo Metastasis	9 (90%) 1 (10%) –	7 (70%) 3 (30%) –	6 (60%) 2 (20%) 2 (20%)	0.228

Intraoperative features were evaluated. Docking time was higher in the Versius^®^ group: 7 (5–8.2) min vs 5 (4–6) and 5 (5–6.5) min for DaVinci and HUGO™-RAS patients, respectively, though not significantly so (*p* = 0.059). Console time and total operative time were comparable: 58.5 (IQR 38.7–63.5), 58 (IQR 33.2–97.5), and 39 (IQR 33.7–61) min (*p* = 0.393) and 109.5 (IQR 78.7–119.5), 110.5 (IQR 82–140.2), and 104.1 (IQR 72.5–143.5) min (*p* = 0.668) for each group, respectively. Collision rates were also comparable (*p* = 0.189).

No intraoperative or postoperative complications were registered, and postoperative stay was similar between the selected patients (2 days in all groups, *p* = 0.629).

After definitive pathologic evaluation, benign lesions were detected in 9 (90%), 7 (70%), and 6 (60%) patients in DaVinci, HUGO™-RAS, and Versius^®^ groups, respectively. Pheochromocytoma were confirmed in 1 (10%), 3 (30%), and 2 (20%) patients per group, respectively, while 2 metastases were registered among the Versius^®^ patients.

## Discussion

This preliminary study supports the feasibility of RAA performed by means of newer platforms, such as HUGO™-RAS and Versius^®^, compared with well-established DaVinci system, for selected cases of adrenal surgical disease in a high-volume endocrine referral center.

The introduction of robotic technology in clinical practice pointed out several limitations of conventional laparoscopy, increasing the inclination towards facilitating surgical solutions. Improved magnification, wider movement degree, and inferior surgeon’s fatigue represent some of the benefits of robotic approach [22–25]. In this context, after the first description of application of robotic technology to adrenalectomy with AESOP 2000 system in 1999 [20, 26, 27], safety and effectiveness profile of RAA has been mainly based on the DaVinci system, thus becoming the referral platform for robotic procedures in clinical practice [28].

However, while the advantages of the laparoscopic over the open approach, namely inferior postoperative pain and morbidity and shorter hospital stay and functional recovery, are well known and recognized by different studies [8, 29, 30], clinical improvements due to the application of robotic technology have not been clearly assessed yet [22–25]. Indeed, the explicit benefit of RAA over laparoscopic approach to adrenalectomy is still a matter of debate and did not receive general consensus [22, 25]. On the other hand, advantages due to the robotics-related technical facilities have been described when the most challenging cases and patients were addressed to adrenalectomy. In our previously published experience, we reported the maximized efficiency of the surgical procedure in obese patients and in large and hyperfunctioning lesions [10], as also reported by other authors [26, 27]. Moreover, the most recent ESES expert opinion [17] described robotic platforms as potential surgical alternatives to other minimally invasive approaches even for the treatment > 6 cm lesions, due to their superiority in terms of three-dimensional vision, dexterity, and stability, thus minimizing the risk of capsular rupture or positivity of resection status [31], suggesting that RAA may be the preferred technique when a minimally invasive procedure is pursued [31].

These recent inclinations towards the advantages of robotic technology for the most complex cases and the concurrent expire of the DaVinci system patents led to an increased diffusion of newer platforms in clinical practice.

Delving deeper, HUGO™-RAS application to adrenalectomy showed its safety and feasibility in different evidences [10, 12, 21, 32, 33], as also showed in our clinical experience [10, 12, 21]. Preliminary reports of the application of further robotic platforms to adrenalectomy, such as KD-SR-01^®^ (SuZhou Kang Duo Robot Co., Ltd., Suzhou, China) and Senhance (TransEnterix, Durham, North Carolina, US), have been published, proving their safety profile, although basing on case series [34, 35], while the feasibility of Versius^®^ system has been mainly based on urologic and colorectal surgical procedures [36, 37].

With regard to the primary outcome of the study, in our patients’ population, no postoperative complications were registered. Due to the lack of comparative studies between the different robotic platforms application to adrenalectomy so far, the main reported postoperative complications rate (up to 6.8% [25]) is to relate to comparison between RAA and laparoscopic procedures [1, 22, 25, 38–40], in line with our experience. The main reported postoperative complications after RAA consist in hemorrhage and bleeding (3–4%) [41, 42], hematoma (0.7%) [11], injuries of adjacent organs (3%), wound infection, ileus, atelectasis, and pleural effusion [1, 22, 25, 38–40]. Several authors reported inferior bleeding rates after RAA, thus reflecting in shorter hospitalization [1, 8, 39, 40, 43]. Overall, postoperative morbidity and mortality are reported to be similar when conventional laparoscopic approach was compared with RAA [40].

In this context, we would like to underline that in literature, the reported risk factors for postoperative complications after RAA mainly consist in tumor size, lesion suspicious for malignancy, and lack of completion of the learning curve [40]. The homogeneity in our study population enabled us to point out such considerations. Indeed, patients’ and lesions’ preoperative features did not significantly differ among the three groups. BMI was only slightly higher in the DaVinci group: 27.9 (IQR 25-4-30.1) kg/m^2^ vs 24.9 (IQR 22.6–28.3) and 26.1 (IQR 25.2–19.3) kg/m^2^ for HUGO™-RAS and Versius^®^. ASA score, previous abdominal surgery and hyperfunction pattern were also comparable. Lesion’s size was lower in the Versius^®^ group, but not statistically so (*p* = 0.236). We believe that the lacking completion of the learning curve is also not to consider a potential confounding factor of increased postoperative complications in our analysis. Indeed, the overall adrenal caseload of our institution during the study period was more than 250 adrenalectomies, including almost 50 RAAs, with an annual volume of adrenalectomies of approximately 90 cases in the last year. Moreover, all robotic procedures in this series were performed by the same expert endocrine surgeon.

Intraoperative complications, consisting of one of the secondary outcomes of our study, were not registered in our patients’ population. This aspect is also in line with the literature reports, showing a lower rate of intraoperative complications in patients undergoing RAA compared to conventional minimally invasive approach, especially in terms of blood loss [30]. Once again, such evidence may be explained with the practical facility of robotic technology, allowing for a more precise detection of bleeding sources during the procedure [24, 30]. In this view, better intraoperative visualization and finer ability to achieve hemostasis leads to avoid further intraoperative damages [30, 49].

Operative time of robotic procedures is known to be longer compared to laparoscopic adrenalectomy at the initial phases of application, with some studies detecting docking steps as main responsible for such difference [5]. In our analysis, operative time, docking time, and console time were similar after comparing the use of each of the three platforms (*p* = 0.668). Overall, total operative time in our experience was comparable to other authors reported results [44, 45]. Nonetheless, extensive expertise with laparoscopic surgery and wide exposure to robotic technology have been reported as relevant variables on total operative time [41, 46]. Moreover, only surgical procedures performed in the same period with each of the three platforms were considered, to overcome potential biases due to the analysis of patients treated with RAA in our preliminary phases of our robotic experience. It is to highlight that several authors [30, 47, 48] reported even shorter operative time when RAA was compared to laparoscopic adrenalectomy, due to a more efficient dissection related to robotic instruments articulation and stability. Agcaoglu et al. [45] also reported similar outcomes, underlining the decreased required time for hemostasis after dissection thanks to increased precision during surgery [30].

Furthermore, docking system may also relate to disparities in terms of operative time when different robotic platforms are compared. Indeed, while DaVinci platform alignment is automatic to facilitate the docking steps, both HUGO™-RAS and Versius^®^ systems do not have a memory for the docking for each procedure, thus requiring a manual configuration for every surgery, explaining longer docking time compared to DaVinci platform [21]. Details of the main advantages and disadvantages of the three different robotic platforms are reported in Table 2.

**Table 2:** Advantages and disadvantages of each robotic platform

	Advantages	Disadvantages
DaVinci Xi	Automatic docking Indocyanine green visualization	High costs Second console required to allow others to visualize the surgical procedure
HUGO™-RAS	Open console Indocyanine green visualization	Manual docking Unavailability of specific energy devices
Versius^®^	High similarity to laparoscopic instruments Indocyanine green visualization	Manual docking Unavailability of specific energy devices Inferior length of the instruments

This study has the merit of being the first one, in our knowledge, comparing the application of DaVinci, HUGO™-RAS and Versius^®^ platforms to adrenalectomy in a high-volume endocrine referral center, with consolidated experience in treatment of adrenal diseases. Moreover, patients’ selection in terms of preoperative features, operated over the same period, was performed to overcome potential biases due to wider personal experience with well-established DaVinci technology. In this context, the homogeneity of reported data may be considered one of the strengths of our analysis.

This study also presents some limitations, such as the retrospective non-randomized nature of the analysis and the limited study population, though mostly related to the availability of the different platforms in clinical practice. As a consequence, further more detailed subgroup analyses, potentially straightening our results, could not be performed.

Although the economic analysis does not represent one of the study endpoints, it is to underline that costs still represent the main hurdle to the worldwide diffusion of robotic technologies, with reports in literature reporting costs more than two times higher compared to conventional laparoscopic adrenalectomy [42, 50]. Even though some authors [12] advocated medical devices expenses as the main cause of the robot procedures-related increased costs, several factors are to take into account when economic sustainability of these technologies is evaluated. Indeed, financial reimbursement model may significantly vary among the different countries and even within the same one, thus explaining the wide heterogeneity of the available evidence concerning this subject.

The introduction of new robotic technologies probably may lead to competitive pricing strategies, hence to significant shift in the current economic balance with the longstanding monopoly of the established DaVinci Surgical System. The economic competition may consequently lead to a further diffusion of valuable alternatives in clinical practice. As a result, the availability of a wider patients' population undergoing surgical procedures by means of different platforms will probably allow for perspective or randomized studies, which may more consistently validate our preliminary results concerning the comparison of different robotic systems.

## Data Availability

The data presented in this study are available on request from the corresponding author.
